# Involvement of Basal Ganglia Network in Motor Disabilities Induced by Typical Antipsychotics

**DOI:** 10.1371/journal.pone.0006208

**Published:** 2009-07-09

**Authors:** Jonathan Chetrit, Bérangère Ballion, Steeve Laquitaine, Pauline Belujon, Stéphanie Morin, Anne Taupignon, Bernard Bioulac, Christian E. Gross, Abdelhamid Benazzouz

**Affiliations:** 1 Université de Bordeaux, Bordeaux, France; 2 Centre Nationale de la Recherche Scientifique, Unité Mixte de Recherche 5227 (CNRS UMR 5227), Bordeaux, France; 3 Centre Hospitalier Universitaire de Bordeaux, Bordeaux, France; University of Nebraska, United States of America

## Abstract

**Background:**

Clinical treatments with typical antipsychotic drugs (APDs) are accompanied by extrapyramidal motor side-effects (EPS) such as hypokinesia and catalepsy. As little is known about electrophysiological substrates of such motor disturbances, we investigated the effects of a typical APD, α-flupentixol, on the motor behavior and the neuronal activity of the whole basal ganglia nuclei in the rat.

**Methods and Findings:**

The motor behavior was examined by the open field actimeter and the neuronal activity of basal ganglia nuclei was investigated using extracellular single unit recordings on urethane anesthetized rats. We show that α-flupentixol induced EPS paralleled by a decrease in the firing rate and a disorganization of the firing pattern in both substantia nigra *pars reticulata* (SNr) and subthalamic nucleus (STN). Furthermore, α-flupentixol induced an increase in the firing rate of globus pallidus (GP) neurons. In the striatum, we recorded two populations of medium spiny neurons (MSNs) after their antidromic identification. At basal level, both striato-pallidal and striato-nigral MSNs were found to be unaffected by α-flupentixol. However, during electrical cortico-striatal activation only striato-pallidal, but not striato-nigral, MSNs were found to be inhibited by α-flupentixol. Together, our results suggest that the changes in STN and SNr neuronal activity are a consequence of increased neuronal activity of globus pallidus (GP). Indeed, after selective GP lesion, α-flupentixol failed to induce EPS and to alter STN neuronal activity.

**Conclusion:**

Our study reports strong evidence to show that hypokinesia and catalepsy induced by α-flupentixol are triggered by dramatic changes occurring in basal ganglia network. We provide new insight into the key role of GP in the pathophysiology of APD-induced EPS suggesting that the GP can be considered as a potential target for the treatment of EPS.

## Introduction

Clinical treatments with typical antipsychotic drugs (APDs) are accompanied by extrapyramidal motor side-effects (EPS). These side-effects are supposed to be due to the dopaminergic antagonist properties of APDs [1], [2]. Accordingly, acute blockade of dopamine transmission in rodent induce motor disturbances such as hypokinesia and catalepsy. Most studies addressing possible sites mediating EPS have been focused on the expression of immediate early genes within the basal ganglia network [3], [4], however, little is known about the relationship between EPS and the changes in basal ganglia neuronal activity.

The basal ganglia comprise a group of highly interconnected subcortical nuclei that are intimately involved in the control of movement. The striatum represents the principal input structure of the basal ganglia and the substantia nigra *pars reticulata* (SNr) and the entopeduncular nucleus (the equivalent of the internal globus pallidus in primate) constitute the major output structures of the basal ganglia projecting to the thalamus and the brainstem. According to the current model of basal ganglia functional organisation [5], [6], input and output structures are linked via a monosynaptic direct pathway and a polysynaptic indirect pathway that involves the globus pallidus (GP) and the subthalamic nucleus (STN). These two pathways arise from two different populations of medium spiny neurons (MSNs) in the striatum (for review, [7]) [8]. The direct pathway is formed by GABAergic striato-nigral MSNs expressing the peptides substance P, dynorphin, neurokinin A and neurotensin and the indirect pathway is formed by GABAergic striato-pallidal MSNs expressing enkephalin and neurokinin B. These two direct and indirect pathways are differentially regulated by dopamine through their respective expression of the excitatory D1 and inhibitory D2 receptors.

Since typical APDs induce EPS and that the relationship between these motor disabilities and changes in basal ganglia neuronal activity has not been clearly established, the present study aimed to investigate the effects of a typical APD, α-flupentixol, upon (i) behavioral motor activity and catalepsy measured by an open field and the bar test respectively, and (ii) extracellular single unit recordings of basal ganglia nuclei including the GP, the STN, the SNr and antidromically identified MSNs of the striatum.

## Materials and Methods

### Animals

Adult male Wistar rats, weighing 280–380 g were used for behavioral and *in vivo* electrophysiological experiments. Animals were provided by the “Centre d'Elevage Depré” (Saint Doulchard, France) and arrived at least one week before use. They were housed five per cage under artificial conditions of light (light/dark cycle, light on at 7:00 a.m.), temperature (24°C), and humidity (45%) with food and water available *ad libitum*. All animal experiments were carried out in accordance with the European Communities Council Directive of 24 November 1986 (86/609/EEC).

### Lesion of the globus pallidus

As previously described [9], [10], rats were placed in a stereotaxic frame (Kopf, Unimecanique, France) under chloral hydrate anaesthesia (400 mg/kg, i.p., Sigma). Each animal received a unilateral injection of 0.4 µl ibotenic acid (Sigma, 8 mg/ml in sterile PBS) into the globus pallidus at coordinates 1 mm posterior to bregma, 3 mm lateral to the midline, and 6.5 mm below the skull according to the brain atlas of Paxinos and Watson [11]. The ibotenic acid injection was made over a 1 minute period of time using a digital 10 µl Hamilton microsyringe. At the end of the injection, the syringe needle was left in place for an additional 10 minutes and then withdrawn slowly to prevent reflux of the solution.

#### Histological validation of the GP lesion

After completion of experiments, fresh-frozen brains were cryostat-cut into coronal 20 µm sections for further validation of the extent of lesion using Cresyl violet staining, as previously described [9], [10]. Only those brains in which a total or subtotal lesion of GP was observed were used for data analysis, whereas animals with lesion outside of the GP were excluded from the study.

### Drug

α-flupentixol (2-[4-[3-[2-(trifluoromethyl)thioxanthen-9-ylidene]propyl]piperazin-1-yl]ethanol) was purchased from Sigma (Saint-Quentin Fallavier, France) and dissolved in NaCl 0.9%. The dose of 0.3 mg/kg for intraperitoneal (i.p.) injection of α-flupentixol was selected on the basis of a detailed litterature search, showing a significant effect on spontaneous motor activity in the rat [12], [13].

### Evaluation of motor activity (Open-field)

Spontaneous horizontal motor activity, vertical activity (or rearing) and stereotyped movements (grooming, washing, and other movements with low amplitudes and independant of locomotor activity) were measured using a photoelectric actimeter (Actitrack, Panlab, S.L., Barcelona, Spain), as previously described [14], [15]. Briefly, the apparatus consisted of a transparent cage that was connected to a photoelectric cell. Light beams detected movement and the total motor activity of each rat was recorded. All testings in the actimeter were done in an isolated room between 8:00 a.m. and 1:00 p.m. The protocol consisted of three phases:

(A) Habituation: spontaneous motor activity was recorded during four consecutive days in two consecutive sessions of 10 minutes, 50 minutes after i.p. injection of NaCl 0.9%. The first 10 minutes session was used to establish the daily habituation. Only the motor activity recorded during the second session of 10 minutes was used for data analysis. Between-session habituation was analysed by comparing behavior in the actimeter on day 3 *vs* that on day 4.

(B) Challenge: α-flupentixol (0.3 mg/kg, i.p.) was injected on day 5, 50 minutes prior the mesurement of motor activity.

(C) Post-challenge: one day after α-flupentixol injection, the rats were re-exposed to the actimeter 50 minutes after i.p. injection of NaCl 0.9%.

Statistical analyses were done using Prism (GraphPad Software, San Diego, CA). Values (counts per 10 minutes) were compared using the Wilcoxon matched-pairs signed ranks test.

### Evaluation of catalepsy scores (bar test)

The standard bar test was used to determine the intensity of catalepsy every 20 minutes after drug injection for 180 minutes as previously reported [16], [17]. Both of the rat's forepaws were placed on a horizontal bar (diameter, 0.7 cm), which was 9 cm above the surface. We recorded the time from placing the forepaws to the first complete removal of one of them from the support bar with a cutoff time of 120 seconds, which is termed here as descent latency. For rats with ibotenic acid lesion of the GP, the catalepsy tests were made 1 week after surgery.

### Extracellular single unit recordings

Extracellular single-unit recordings were made in rats anesthetized with urethane (1.2 g/kg, i.p.) as previously reported [15], [18]. For rats with ibotenic acid lesion of the GP, recordings were made two weeks after surgery. For all basal ganglia nuclei recordings were made with single glass micropipette electrodes (impedance: 8–12 MΩ; aperture ∼0.5 µm), which were filled with 4% Pontamine sky blue in 3 M NaCl, then placed into the right target structure according to the coordinates given in the brain atlas [11] (for GP, AP: −1 mm posterior to bregma, L: −3 mm from the midline, D: 5.7–7.3 mm from the dura; for STN, AP: −3.8 mm, L: −2.5 mm, D: 6.8–8.2 mm; for SNr, AP: −5.3 mm, L: −2.5 mm, D: 7.5–8.6 mm; for striatum, AP: +1.2 mm, L: −3 mm, D: 3.5–5.5 mm). Extracellular neuronal activity was amplified, bandpass-filtered (300–3000 Hz) using a preamplifier (Neurolog, Digitimer, UK), displayed on an oscilloscope, transferred *via* a Powerlab interface (AD Instruments, Charlotte, NC, USA) to a computer equipped with Chart 5 software (AD Instruments, Charlotte, NC, USA). Only neuronal activity with a signal-to-noise ratio >3∶1 was recorded and used for further investigation. Basal firing of neurons was recorded for 20 minutes before drug injection to ascertain the stability of the discharge activity. α-flupentixol or NaCl 0.9% (used as control) was then injected intraperitonealy. At the end of each session, the recording site was marked by electrophoretic injection (Iso DAM 80, WPI, Hertfordshire, UK) of Pontamine sky blue through the micropipette at a negative current of 20 µA for 7 minutes.

For recordings in the striatum, four types of neurons can be identified. Tonically active neurons are characterized by their unique, regularly spaced, spontaneous activity between 2 and 6 Hz. These neurons are presumed to be cholinergic interneurons. Fast spiking GABA interneurons (FSI) are characterized by their spike duration, which is briefer than that of all other striatal neurons, and by the fact that they respond with brief burst of 2 to 5 action potentials to supra threshold cortical stimulation [19], [20]. These two populations of striatal interneurons were not further studied here. Our interest was focused on the two other populations named medium spiny neurons (MSNs), which send efferents to the GP and the SNr. Single MSNs were extracellularly recorded from the rostrolateral striatum in parallel with cortical EEG recordings as previously described [20]. In urethane-anesthetized rats the dominant cortical state exhibits a rhythmic activity characterized by slow waves of large amplitude at a frequency close to 1 Hz. Antidromic stimulation of the SNr was used to identify MSNs [20]. Concentric bipolar electrodes (SNEX-100, Rhodes Medical Instruments, Summerland, CA 93067, USA) were implanted in the rostral pole of SNr (2.4 mm to medial line, 4.9 mm caudal to bregma and 8.0 below the cortical surface). Striato-nigral neurons exhibited antidromic responses to SNr stimulation according to the following criteria: (1) constant latency of spike response, (2) all-or-none property of the spike response when the strength of the stimulation was adjusted just above, or just below, threshold, and (3) collision of the antidromic spikes with orthodromic spikes. Because most striatal neurons are silent or discharge at very low firing rate, the orthodromic spike required for the collision test was evoked by cortical stimulation and the SNr stimulation was triggered by the spike evoked by cortical stimulation with a delay of 30 or 3 ms, alternately. MSNs, which did not exhibit the features of striatal interneurons and which did not exhibit antidromic responses to SNr stimulation were qualified as striato-pallidal neurons [19]. By using double labeling, these authors have already shown that these MSNs express the mRNA coding for ENK, a recognized feature of striato-pallidal neurons [19]. In order to test the dynamic response of MSNs to cortical input we used paired cortical stimulations at 100 ms intervals as previously described [19].

In all experiments described here cortical EEG was continuously monitored to assert that the recorded animal was actually in the slow wave state. If disruption of the slow waves spontaneously occurred, the slow wave state was restored with additional i.p. injection of urethane (15% of the initial dose). α-flupentixol (0.3 mg/kg) or saline were administered i.p. only once per rat.

#### Validation of the recording sites

After completion of experiments, animals were sacrificed by an overdose of urethane, the brains removed, frozen in isopentane at −45°C and stored at −80°C. Fresh-frozen brains were cryostat-cut into 20 µm coronal sections for further validation of the location of recording track into the GP, STN and SNr as previously described [15], [18]. To this aim, acetylcholine esterase staining was used to contrast structures and make more easy to determine the location of the Pontamine sky blue dots marking the recording sites in each structure. Only those brains in which the location of the Pontamine sky blue dot was clearly visible in the target structure were used for data analysis.

#### Data analysis

The activity of each neuron was analyzed with a spike discriminator using a spike histogram program (AD Instruments, Charlotte, NC, USA) and firing parameters (firing rates and interspike intervals with a bin of 5 msec) were calculated using Neuroexplorer program (AlphaOmega, Nazareth, Israel). Firing patterns were analyzed using the method based on the determination of density histogram developed by Kaneoke and Vitek [21] as previously described [18], [22]. The coefficient of variation of the interspike interval, which determines the regularity of neuronal firing, is defined as the ratio of the standard deviation to the mean interspike interval. For each parameter, the values obtained before and after α-flupentixol injection were compared using a one-way ANOVA with repeated measures followed by the Dunnett's test (post-hoc).

## Results

### Effects of α-flupentixol on motor activity and catalepsy

**Figure 1:**
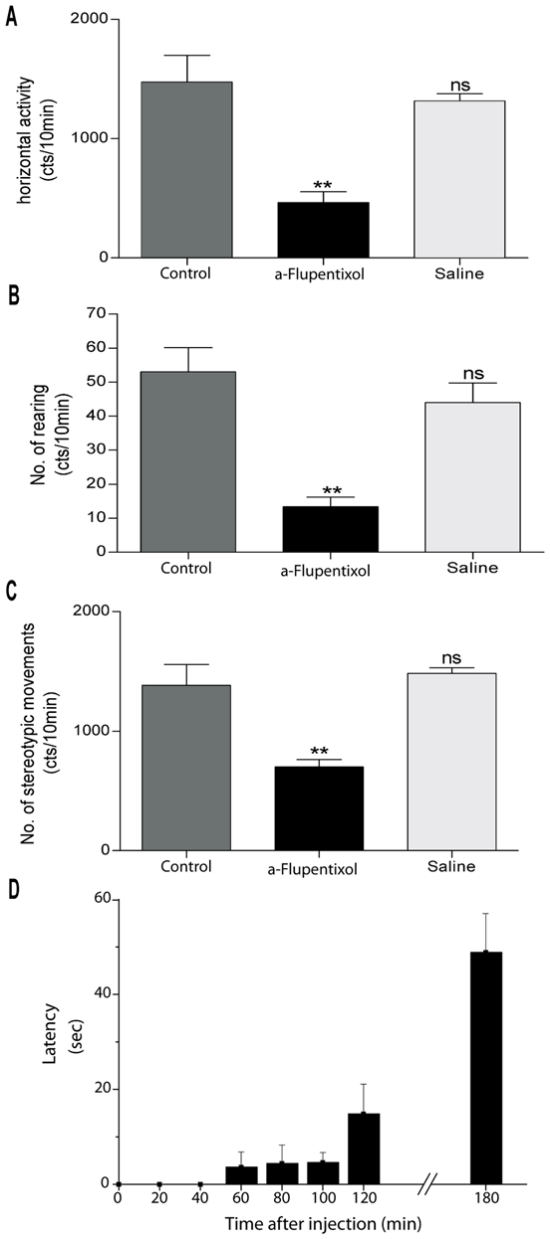
α-flupentixol induces hypomotor activity and catalepsy. (A–C) Histograms illustrating the scored motor activity recorded during the second 10 minutes session fifty minutes after the injection of saline (control) or α-flupentixol (0.3 mg/kg) or saline on day 4, 5 and 6 respectively. Note that α-flupentixol induced a significant decrease of the scored horizontal activity (A), vertical activity (B) and stereotypic movements (C) in comparison with controls (n = 10 rats) (Wilcoxon test, **p<0.01). No significant difference was observed after saline injection on day 6 in comparison with controls (Wilcoxon test, ns: no-significant, p>0.05). (D) Histogram displaying the evolution of catalepsy induced by the injection of α-flupentixol. Measurement of the latency from paw placement until the first complete removal of one paw from the bar during the catalepsy test. Values are presented as the mean±S.E.M. cts/10 min = counts/10 minutes.

As previously reported [15], using an open field photoelectric actimeter, the behavior of the animals was stable after three days, indicating that the rats were habituated to their test environment. Values are presented as mean of counts per 10 minutes±S.E.M. Results reported as “control” on figure 1 correspond to the measure on day 4 after i.p. saline injection. α-flupentixol (0.3 mg/kg) injection significantly decreased horizontal activity by 69% (463.8±92.4 vs 1474.5±225.5 in control), rearing by 75% (13.4±2.8 vs 53.0±7.1 in control) and stereotypy by 49% (702.6±62.4 vs 1388.0±172.8 in control) in comparison with control saline injection performed on day 4 (Wilcoxon test, *p*<0.01, *n* = 10). A post-challenge test was performed on day 6 after i.p. saline injection to confirm the specific effect of α-flupentixol. No significant difference was observed in comparison with the motor activity measured on day 4 after saline injection (Fig. 1A–C) confirming that the motor activity returned to the basal level.

The cataleptic effect of α-flupentixol injection was measured using the bar test. Catalepsy started 60 minutes after i.p. injection of α-flupentixol (3.6±3.1 sec descent latency) and was maximal 180 minutes after (48.8±10.1 sec descent latency) (Fig. 1D).

### Effects of α-flupentixol on SNr neuronal activity

**Figure 2:**
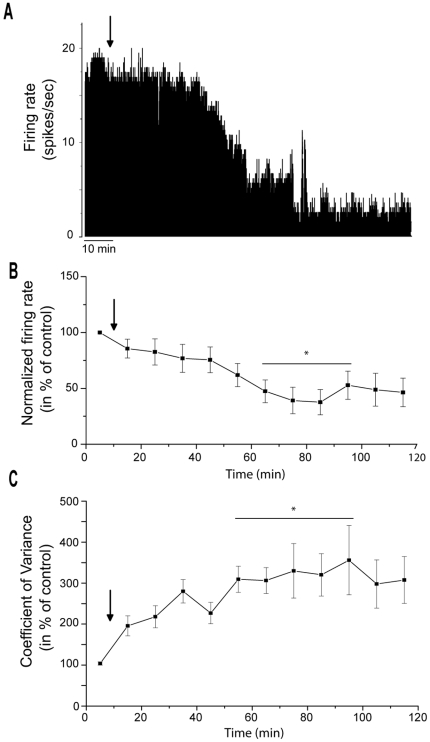
α-flupentixol induces changes of the electrical activity of substantia nigra *pars reticulata* neurons. (A) A representative example of a firing rate histogram showing the inhibitory effect of α-flupentixol on SNr neuronal activity. (B) α-flupentixol decreases the firing rate of the majority of SNr neurons tested (n = 10/14), and (C) increases the coefficient of variance of the interspike interval (ISI) (One-way ANOVA with repeated measures followed by the Dunnett's test, *p<0.05). Arrows indicate the time at which α-flupentixol was administred.

**Figure 3:**
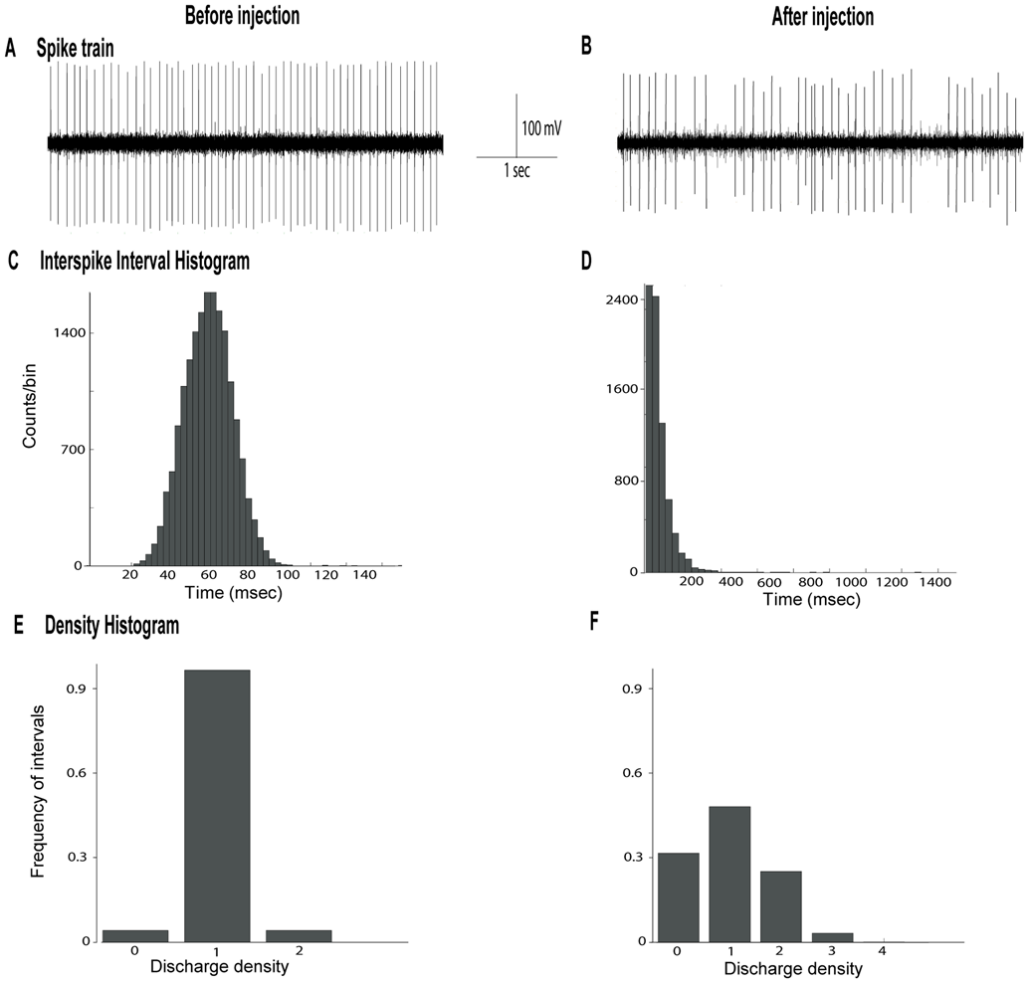
α-flupentixol alters the firing patterns of substantia nigra *pars reticulata* neurons. (A and B) Sections of extracellular recordings of action potentials, before and after injection respectively, showing that α-flupentixol reduced the firing rate and made the pattern irregular. (C and D) Interspike interval histograms, (E and F) density histograms, respectively before and after α-flupentixol injection confirming the disorganization of the firing pattern induced by α-flupentixol in the same neuron.

As the SNr represents the major output structure of the basal ganglia in the rat, playing a key role in the motor control, we determined whether systemic administration of α-flupentixol induced changes in its neuronal activity (n = 14 neurons in 14 rats). The spontaneous firing rate of SNr neurons ranged from 7.3 to 31.3 spikes/sec with a mean of 23.8±2.0 spikes/sec. α-flupentixol induced a significant decrease in the firing rate [F = 4.73, *p*<0.0001] (−55.7±12.5%, at 85 minutes, *p*<0.05) of the majority of SNr neurons (n = 10/14) (Fig. 2 and 3). This effect occurred 60 minutes after the injection and no recovery to the basal level was observed (Fig. 2A and 2B). The four other neurons did not show any modification of their firing activity after α-flupentixol injection. As shown in figure 2C, coefficient of variance of the intespike intervals (ISI) significantly increased after i.p. injection of α-flupentixol [F = 2.79, *p* = 0.004] (308.8±54.0% at 75 minutes, *p*<0.05) traducing a disorganization of the firing pattern of SNr neurons (Fig. 3A and 3B), which was maintained for more than 2 hours. This abnormal pattern of SNr neurons was confirmed by the Kaneoke and Vitek method based on the analysis of the ISI (Fig. 3C and 3D) and the density (Fig. 3E and 3F) histograms, which showed clear differences before and after α-flupentixol injection in all the 10 neurons.

### Effects of α-flupentixol on STN neuronal activity

**Figure 4:**
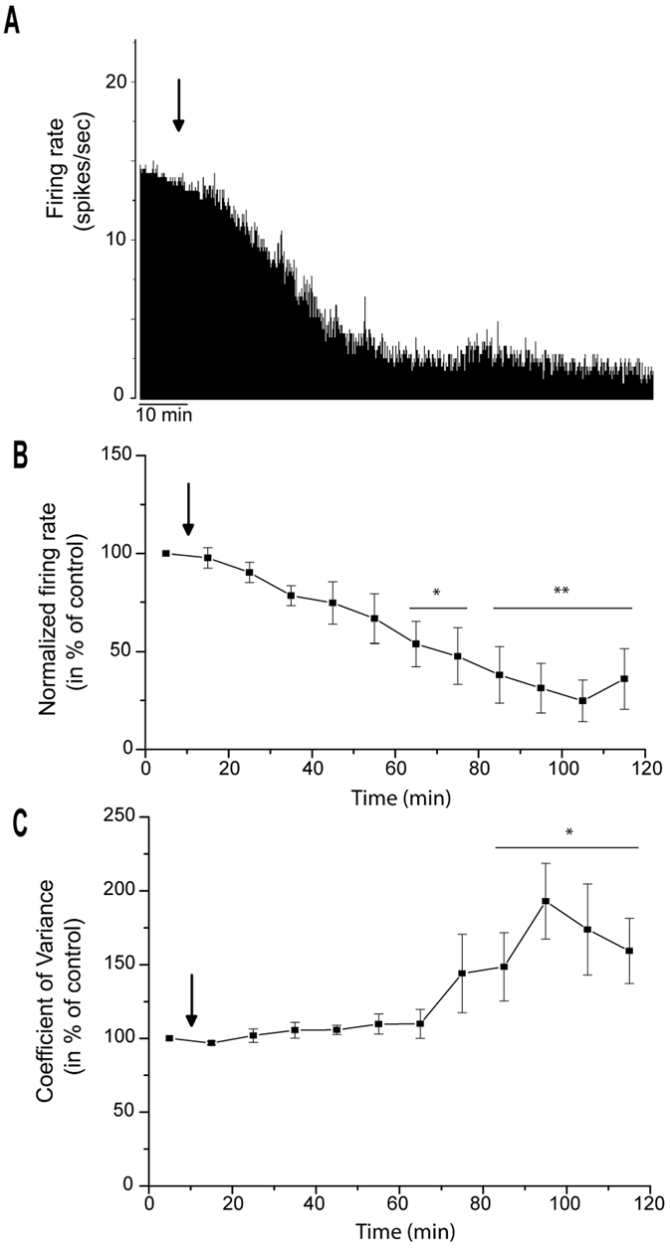
α-flupentixol induces changes of electrical activity of subthalamic nucleus neurons. (A) A representative example of a firing rate histogram showing the inhibitory effect of α-flupentixol on STN neuronal activity. (B) α-flupentixol decreases the firing rate of all STN neurons tested (n = 9/9), and (C) increases the coefficient of variance of the interspike interval (ISI) (One-way ANOVA with repeated measures followed by the Dunnett's test, *p<0.05, **p<0.01). Arrows indicate the time at which α-flupentixol was administred.

**Figure 5:**
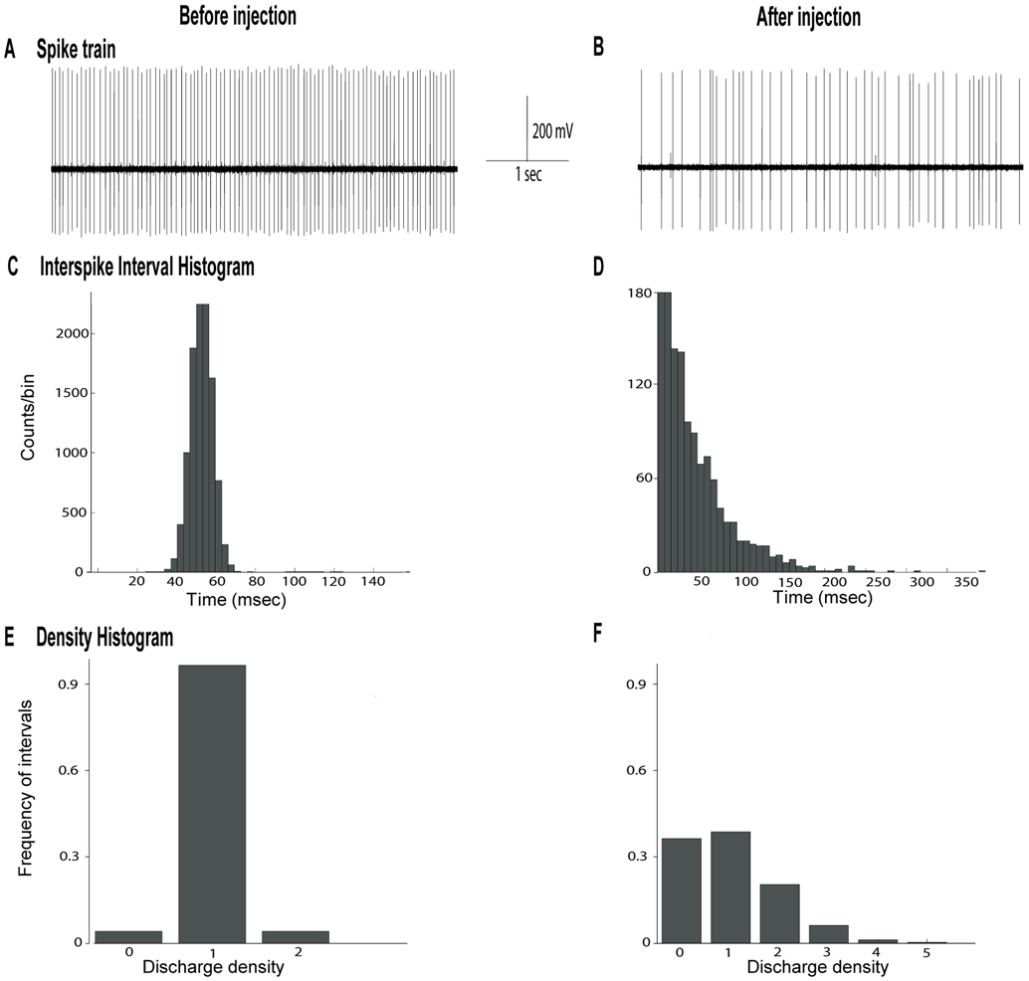
α-flupentixol alters the firing patterns of subthalamic nucleus neurons. (A and B) Sections of extracellular recordings of action potentials, before and after injection respectively, showing that α-flupentixol reduced the firing rate and made the pattern irregular. (C and D) Interspike interval histograms, (E and F) density histograms, respectively before and after α-flupentixol injection confirming the disorganization of the firing pattern induced by α-flupentixol in the same neuron.

The STN, which is the only glutamatergic nucleus of the basal ganglia network, is considered as a driving force exerting an excitatory tonic influence on the SNr. The spontaneous firing rate of STN neurons ranged from 11.7 to 16.9 spikes/sec with a mean of 14.6±1.9 spikes/sec. Responses of STN neurons to α-flupentixol are shown in figures 4 and 5. As for the SNr, similar changes were recorded in the STN. α-flupentixol injection (0.3 mg/kg, i.p.) provoked a clear inhibitory effect in STN neurons [F = 4.68, *p*<0.0001]. All neurons (*n* = 9/9, n = 9 rats) showed a significant decrease in their firing rate (−47.0±5.8%, at 75 minutes, *p*<0.05) with a maximal effect at 115 minutes (−75.5±8.9%, *p*<0.01). This effect occurred 60 minutes after the injection and no complete recovery to the basal level was observed (Fig. 4A and 4B). All neurons showed a significant increase of the coefficient of variance of the ISI [F = 2.88, *p* = 0.002] (192.9±28.1% at 100 minutes, p<0.05, n = 9) suggesting that α-flupentixol induced a disorganization of the firing pattern of STN neurons (Fig. 4C and Fig. 5A and 5B). This was confirmed by the Kaneoke and Vitek method based on the analysis of the ISI (Fig. 5C and 5D) and the density (Fig. 5E and 5F) histograms, which showed clear differences before and after α-flupentixol injection.

We suspected that the changes in SNr and STN neuronal activities originated at an upstream location. As a part of the indirect pathway, GP exerts a tonic inhibitory influence on both STN and SNr.

### Effects of α-flupentixol on GP neuronal activity

**Figure 6:**
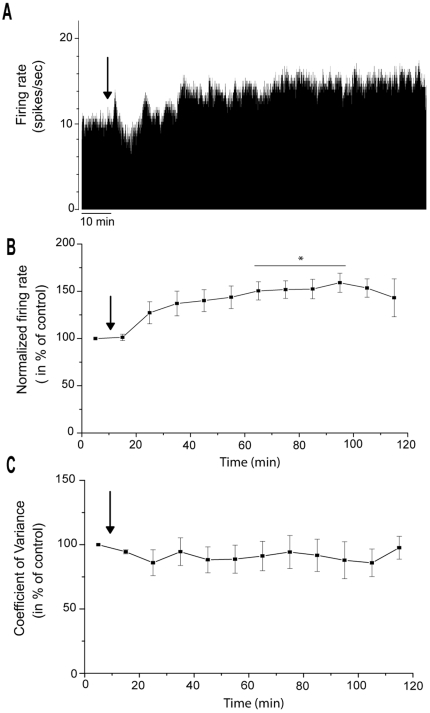
α-flupentixol induces changes of electrical activity of globus pallidus neurons. (A) A representative example of a firing rate histogram showing the excitatory effect of α-flupentixol on GP neuronal activity. (B) α-flupentixol increases the firing rate of all GP neurons tested (n = 9/9), without any modification of the coefficient of variance of the interspike interval (ISI) (C) (One-way ANOVA with repeated measures followed by the Dunnett's test, *p<0.05). Arrows indicate the time at which α-flupentixol was administred.

**Figure 7:**
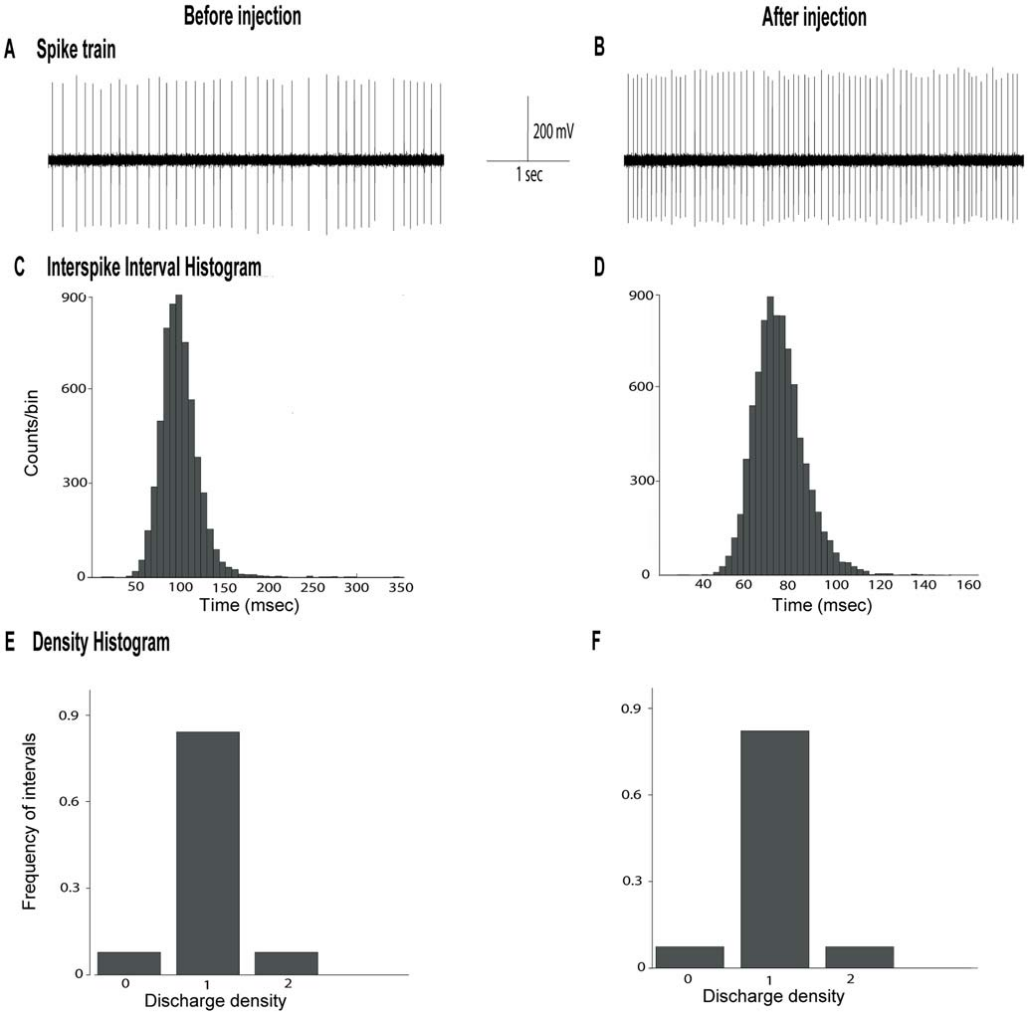
α-flupentixol does not alter the firing patterns of globus pallidus neurons *in vivo.* (A and B) Sections of extracellular recordings before and after injection respectively, showing that α-flupentixol increased the firing rate without modifying the firing pattern. (C and D) Interspike interval histograms, (E and F) density histograms, respectively before and after α-flupentixol injection confirming the absence of changes of the firing pattern in the same neuron.

The spontaneous firing rate of GP neurons ranged from 9.7 to 24.1 spikes/sec. with a mean of 13.4±2.1 spikes/sec. Responses of GP neurons to α-flupentixol are shown in figure 6 and 7. α-flupentixol injection (0.3 mg/kg) induced a clear excitatory effect on GP neurons [F = 4.08, *p* = 0.0003] (Fig. 6 and 7). All of the tested neurons (*n* = 9/9, n = 9 rats) showed a significant increase in their firing rate (+50.4±10.5% at 65 minutes, *p*<0.05) in comparison with the basal level. The effect was maintained for more than two hours (Fig. 6A–B). No change in the coefficient of variance of the ISI was observed [F = 0.20, *p* = 0.99] (Fig. 6C) showing that the regularity of the pattern was not altered (Fig.7A and 7B). This result was confirmed by the Kaneoke and Vitek method based on the analysis of the ISI (Fig. 7C and 7D) and the density (Fig. 7E and 7F) histograms, which showed no differences before and after α-flupentixol injection.

### Effects of α-flupentixol on MSNs of the striatum

**Figure 8:**
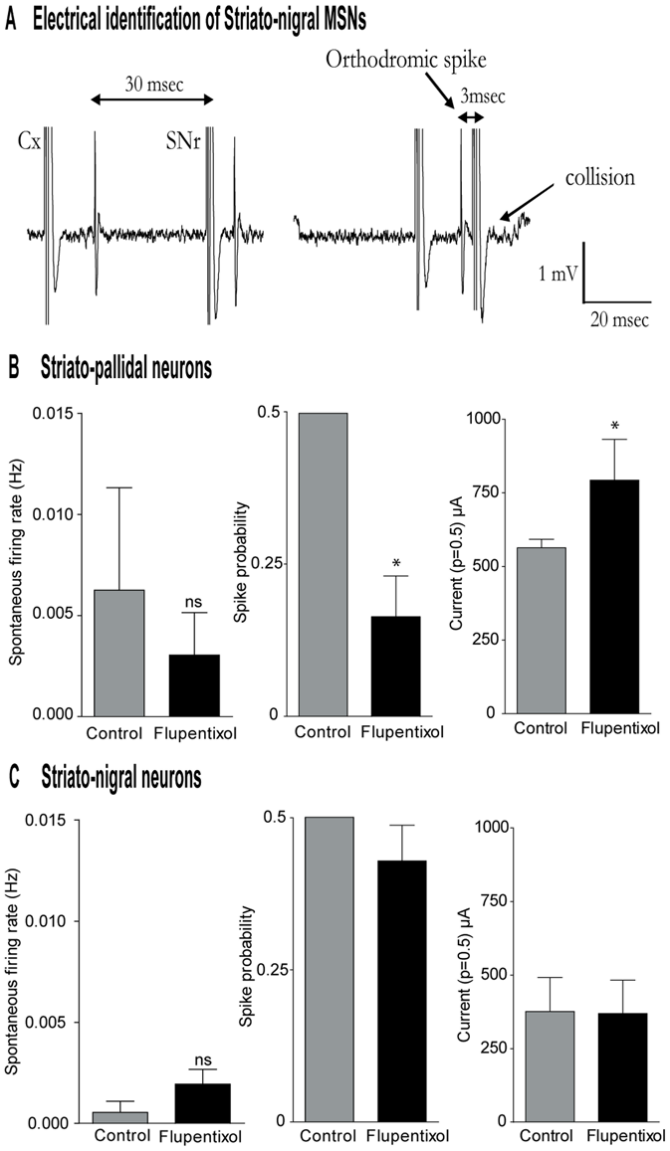
Responses of identified striatal MSNs to α-flupentixol. (A) Electrophysiological identification of striato-nigral neurons. Example of a collision test used to identify a striato-nigral neuron by antidromic activation of the SNr. The SNr stimulation (SNr) was triggered by an orthodromic spike evoked by cortical stimulation (Cx) either with a delay of 30 ms (no collision) or with a delay of 3 ms leading to collision of both spikes. (B) and (C) Responses of striato-pallidal (n = 7) and striato-nigral cells (n = 6) respectively. The left pannel histograms show the spontaneous activity of MSNs, whereas midle and right pannel represent repectively the spike propability response at a fixed cortical stimulating current and the stimulating current required to evoke a spike response with a 50% probability, both, before and after α-flupentixol injection. Note that α-flupentixol did not induce any significant change in the spontaneous firing rate of striato-pallidal MSNs (One way ANOVA, F = 0.28, p = 0.76) nor of striato-nigral MSNs (One way ANOVA, F = 0.66, p = 0.53). However, striato-pallidal MSNs were less responsive to cortical stimulations (p = 0.031) and the stimulating current required to evoke a spike response with a 50% probability was significantly increased after α-flupentixol injection (p = 0.031). Bar histograms indicate mean±S.E.M., *p<0.05, ns: non-significant.

Since the GP receives GABAergic afferents from striato-pallidal MSNs of the striatum and that another neuronal subset, the striato-nigral MSNs, projects to the SNr, we recorded these two populations after their antidromic identification as previously reported [19]. Six neurons (n = 6 rats) were identified as striato-nigral MSNs by antidromic activation of the SNr (Fig. 8A). Seven other MSNs (n = 7 rats) that did not exhibit an antidromic response to SNr stimulation were not classified as interneurons and were identified as striato-pallidal MSNs. Responses to α-flupentixol were recorded between 40 and 120 minutes after i.p. injection. α-flupentixol did not induce any significant change in the spontaneous firing rate of striato-pallidal MSNs (F = 0.28, p = 0.76, n = 6, Fig. 8B left) nor of striato-nigral MSNs (F = 0.66, p = 0.53, n = 7, Fig. 8C left). As most of MSNs are silent or discharge at very low firing rate, (this can explain the high level of error bars of the firing rate histograms) the effect of α-flupentixol was studied on the responses evoked by cortical stimulations. α-flupentixol (0.3 mg/kg) decreased the spike responses evoked in striato-pallidal MSNs by the second pulse of paired cortical stimulations (p = 0.031, Fig. 8B middle). Indeed, the stimulating current required to evoke a spike response with a 50% probability was significantly increased after α-flupentixol injection (p = 0.031, Fig. 8B right). Regarding the striato-nigral MSNs, neither the spike response evoked by cortical stimulations, nor the stimulating current to evoke a spike response with a 50% probability was modified (p = 0.437 and p = 0.843 respectively, Fig. 8C middle and right respectively).

Together, our electrophysiological data show that the indirect pathway, but not the direct pathway, is suspected to play an important role in the manifestation of EPS induced by α-flupentixol. To test this hypothesis, we investigated the effect of selective ibotenic acid lesion of the GP, which is a relay structure in the indirect pathway on α-flupentixol-induced catalepsy and changes in STN neuronal activity.

### Effects of GP lesion on α-flupentixol-induced catalepsy and changes in STN neuronal activity

**Figure 9:**
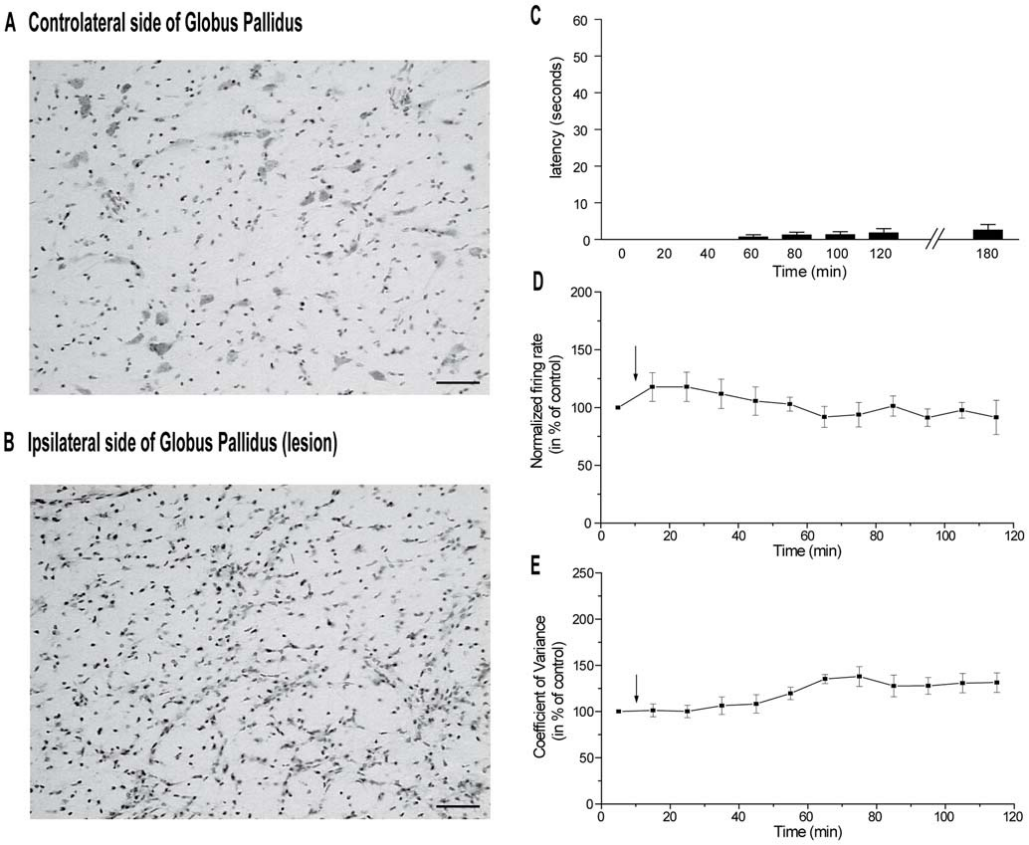
Globus pallidus lesion prevents α-flupentixol-induced catalepsy and changes in the neuronal activity of STN neurons. (A and B) Photomicrographs of the globus pallidus showing the control side (A) and the side which received ibotenic acid injection-induced cell death (B, lesioned side). Scale bar: 50 µm. (C) Histogram illustrating the mean±S.E.M of the latency measured from paw placement until the first complete removal of one paw from the bar during the catalepsy testing (n = 9). Note that ibotenic acid lesion of the globus pallidus prevents α-flupentixol-induced catalepsy. (D and E) Curves illustrating the evolution of the firing rate (D) and the coefficient of variance (E) after the injection of α-flupentixol (arrow) in rats (n = 6) with ibotenic acid lesion of the globus pallidus. Note that this lesion prevented the alteration of the two electrophysiological parameters (One-way ANOVA with repeated measures, p>0.05). Values are presented as the mean±S.E.M.

Cresyl violet staining of coronal sections allowed us to determine the extent of ibotenic acid lesion of GP and only rats with a total or subtotal lesion of the nucleus (Fig. 9A and 9B) were selected for the analysis of behavioral and electrophysiological data. The Bar test measurements showed that after selective ibotenic acid lesions of the GP, α-flupentixol (0.3 mg/kg) failed to induce catalepsy (n = 9 rats) (Fig. 9C). In these animals, the spontaneous firing rate of STN neurons ranged from 10.3 to 38.9 spikes/sec with a mean of 19.8±4.1 spikes/sec (n = 6, in six rats). α-flupentixol (0.3 mg/kg) did not induce any change in the firing rate [F = 0.68, p = 0.77] (Fig. 9D) nor in the firing patterns of STN neurons as shown by the coefficient of variance of the ISI [F = 2.50, p = 0.41] (Fig. 9E).

## Discussion

The present study reports for the first time important experimental evidence for a key role of the basal ganglia network in the manifestation of EPS induced by typical APDs. Indeed, the hypoactivity accompanied by irregular discharge patterns recorded in the STN and the SNr appears to be a specific consequence of increased neuronal activity within the globus pallidus, suggesting that the indirect pathway, but not the direct pathway, plays an important role in the motor effects of α-flupentixol.

α-flupentixol induced catalepsy measured by the bar test with a decrease in the motor activity parameters measured by the open field actimeter. These results confirm the ability of this APD to induce EPS in the rat in agreement with previous reports using α-flupentixol [13] or other typical APDs such as haloperidol [23], raclopride [24], [25] and sulpiride [26]. Several studies suggested that typical APDs have a high EPS liability because they generally induce a more pronounced dopamine D2 receptor occupancy than atypical APDs [27]–[31]. Indeed, haloperidol does not induce catalepsy in D2 knockout mice [32], demonstrating that EPS are specifically due to the blockade of D2 receptors. In line with this, α-flupentixol has been shown to have a higher affinity for D2 than D1 receptors *in vivo* [33].

According to the proposed anatomo-functional model of the motor circuit [5], the blockade of dopamine receptors, which mimics the nigro-striatal denervation in animal models of Parkinson's disease, should result in similar changes in basal ganglia network function, i.e. hyperactivity associated with bursty activity in the major output structure of basal ganglia, the SNr [34]–[36]. This hyperactivity has been interpreted as a result of reduced inhibitory input from the striatum via the direct pathway implicating D1 receptors and a release of the inhibitory input from the striatum to the GP via D2 receptors of the indirect pathway [5], [8], [19], [37]. The decrease in neuronal activity of GP neurons, in turn, induces a disinhibition of STN neurons becoming overactive, and consequently spreads this hyperactivity to SNr neurons. Surprisingly, our electrophysiological findings show that the cascade of changes recorded in different nuclei of the basal ganglia network after the administration of α-flupentixol is dissimilar to those previously found in animal models of parkinsonism.

We show that α-flupentixol did not induce an increase but a dramatic decrease in the firing rate of SNr neurons, which was accompanied by a switch from regular to irregular firing patterns as determined by the coefficient of variance of the ISI and also by the Kaneoke and Vitek method [21]. As the striatum constitutes the major input structure of the basal ganglia network, we recorded the spontaneous activity of identified MSNs at the origin of the direct pathway connecting the striatum to the SNr. Our results show that α-flupentixol was unable to induce any significant change in the spontaneous activity of these identified striato-nigral MSNs and even with cortical stimulations no change in the evoked response was observed. Indeed, it is unlikely that the direct pathway is the origin of changes observed in the SNr. However, in the STN, which is a part of the indirect pathway and at the same time a major excitatory afferent component of the SNr, α-flupentixol induced a dramatic decrease in the firing rate with a disorganization of the firing pattern similar to those recorded in the SNr. From these results, and in accordance with the glutamatergic nature of STN-SNr transmission responsible for the tonic discharge of SNr neurons (for review, [38]), we can postulate that the decrease in the firing rate of STN neurons could result in a deactivation of SNr neurons with a propagation of irregular discharge patterns. This fit with the results of a previous study, which showed that pharmacological blockade of STN neuronal activity resulted in a marked reduction in SNr firing [39]. Moreover, we add evidence that the changes in STN neuronal activity could originate in the GP as we show that α-flupentixol induced an increase in the firing rate of GP neurons. It is well known that STN neurons receive inhibitory GABAergic afferents from the GP [40], [41] and that the increase in the activity of GP neurons could result in an increase of GABA release in this nucleus. These results represent additional evidence for neuroleptic-induced increase of the GP neuronal activity in agreement with other studies using quantitative *in situ* hybridization of GAD_67_ mRNA expression, which is an index of GABAergic activity [42]. The firing rate increase of GP neurons can be also implicated in the decreased neuronal activity recorded in the SNr, since neurons of this nucleus receive dense GABAergic inputs from GP [43], which converge with subthalamo-nigral terminals on the same SNr neurons [44].

While considering how α-flupentixol influences GP activity, it is impossible to ignore the contribution of striatal MSNs. GP neurons receive inhibitory input from striatal MSNs expressing D2 receptors [5], [8], [37]. However, our electrophysiological results show that α-flupentixol did not induce any significant change in the spontaneous activity of striato-pallidal MSNs. Nevertheless, during cortical activation the decrease of the evoked response of striato-pallidal MSNs after the injection of α-flupentixol can reinforce the disinhibition of GP neuronal activity. Thus, at basal conditions, the possible mechanism could be mediated by the direct alteration of dopamine transmission in GP as its neurons express mostly D2 receptors [45]. In agreement with this hypothesis, previous studies have shown that systemic administration of D2 antagonists induced Fos expression, the protein product of the immediate early gene *c-fos*, in the GP by its direct action within GP and not within the striatum [3], [4], [46]. Moreover, dopamine transmission within the GP has been shown to be necessary to achieve motor control. Bilateral blockade of dopamine transmission into the GP induced akinesia and catalepsy [26], [47]. However, the infusion of dopamine in the same nucleus caused improvement of motor deficits in hemiparkinsonian rats [48].

Together, our results suggest that EPSs are correlated with changes in two major structures of the basal ganglia network, the STN and SNr and that these changes may be a consequence of the increased firing rate of GP neurons. This hypothesis was confirmed by our data showing that after selective ibotenic acid lesions of the GP, α-flupentixol failed to induce catalepsy and concomitantly any changes in subthalamic neuronal activity demonstrating the key role of the indirect pathway in the manifestation of EPS. These results are in line with previous studies showing that inactivation of the GP can elicit motor activation or reverse motor hypoactivity. Indeed, GP lesions have been shown to abolish neuroleptic-induced catalepsy [49]–[51].

In conclusion our results emphasize the key role of changes occurring in basal ganglia network implicated in the manifestation of EPS induced by a typical APD, demonstrating that hypokinesia and catalepsy-induced by α-flupentixol are clearly associated with hypoactivity and abnormal irregular patterns of STN and SNr neurons. The changes observed in these two nuclei are not mediated by MSNs of the striatum. Moreover, our study provides new insight into the role of GP, which is a key structure of the indirect pathway, in the pathophysiology of APD-induced EPS and that GP can be considered as a potential target for the treatment of EPS.
